# Using the max-p regions problem algorithm to define regions for energy system modelling

**DOI:** 10.1016/j.mex.2021.101211

**Published:** 2021-01-06

**Authors:** Christian Etienne Fleischer

**Affiliations:** Europa-Universität Flensburg Germany

**Keywords:** Max-p-regions, Spatial aggregation, Renewables

## Abstract

The input data of energy system models are, in many cases, aggregated data used to represent regions of interest. This paper presents a method of using energy-related spatial data and the max-p-regions method to define regions. The method aims to assign areas to regions that are similar in how much they consume, produce and store electricity.

• A spatial dataset of administrative areas for 30 European countries is presented and used to apply the proposed method.
• Use of energy-related spatial data to define regions of energy system model.
• The method uses the heuristic solution of the max-p regions problem to reduce the spatial resolution of energy system models.

## Graphical abstract

**Figure:**
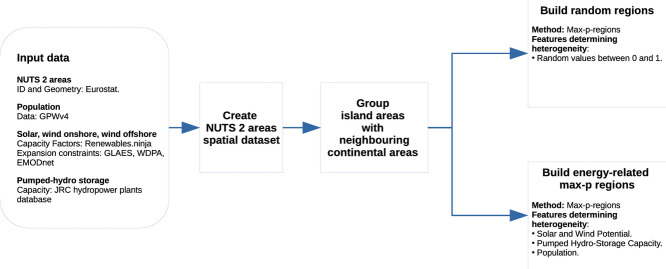

Specifications table

**Table:** 

Subject area:	Energy
More specific subject area:	Energy system modelling
Method name:	Defining regions according to energy-related attributes using the max-p-regions method for energy system modelling.
Name and reference of original method:	The regionalisation method is a combination of the European cluster model method [1] and the spatial aggregation clustering method detailed in [2].
Resource availability:	Data • Nomenclature of territorial units for statistics level 2 geometry [3] • Gridded population of the world version 4 [4] • JRC hydro-power plants database [5] • Normalized maximum power output profiles [21] • Offshore oil and gas pipelines [6] • Protected areas [7] Software • Geospatial Land Availability for Energy Systems framework (GLAES) [8] • pysal [9] • numpy [10] • geopandas [11] • rasterstats [12]

## Method details

 

## Background

Cost optimisation results of energy system models with high penetration of solar and wind are impacted by choice of the spatial resolution of the model. Two documented effects are the increase in transmission expansion cost and the reduction in solar and wind capacity with increasing spatial resolution [18], [19], [20]. The max-p regions method presented in [17] can be effective in minimising these two effects by using several energy-related spatial attributes to define regions during spatial resolution reduction. The max-p regions method builds upon the approached applied by [1] and [2] to define regions. Similar to the clustering method used in [1], the max-p regions method uses multiple energy-related spatial attributes to define similarities between areas but the max-p regions method uses the max-p regions problem algorithm instead of k-means. The clustering algorithm used in [2] uses a combination of both the max-p-regions problem algorithm and k-means but [2] only one spatial attribute to define regions. Two clustering methods are used by [2] as solving the max-p regions problem algorithm as a mixed-integer programming problem with an increasing number of areas can become intractable. The presented max-p regions method uses a heuristic solution to solve the max-p-regions problem presented in [15]. The random regions method described in this article is used in [17] to verify the efficacity of the max-p regions method to define regions that are less impacted by spatial resolution reduction effects.

## Spatial dataset preparation

A georeferenced dataset of the Nomenclature of Territorial Units for Statistics Level 2 NUTS 2 areas for countries in the European Union, plus Norway, Switzerland and the UK is used to apply the method. The spatial dataset is structured using the GeoDataFrame framework of the geopandas tool. The Identification (NUTS_ID), country reference code (CNTR_CODE), and the geometrical information (geometry) of the NUTS 2 areas, extracted from the Eurostat database [3], are added to the dataset. The coordinate reference system (CRS) of the geometry of the areas, is set as EPSG 3035.

Next, the population in the NUTS 2 areas is added to the dataset. The population values are extracted from the gridded population of the world version 4 (GPWv4) of 2015 [4], using the NUTS 2 area geometry and the *zonal_stats* function in the rasterstats python package [12]. The CRS of the GPWv4 GeoTIFF file is WGS84, and therefore the NUTS 2 areas geometry is converted to the same CRS before applying the *zonal_stats* function. The geometries of the NUTS 2 areas dataset is then transformed back to European Petroleum Survey Group (EPSG) 3035.

The sum of the electricity storage capacity of pumped storage hydropower plants within the NUTS 2 areas are added to the spatial dataset. The information on pumped storage hydropower plants is from the Joint Research Centre (JRC) hydropower plants database [5]. The JRC hydropower plants database pumped storage hydropower plants are labelled HPHS in the database type column. The longitude and latitude location values of the hydropower plant database are in CRS EPSG 4326 and therefore are first converted to CRS EPSG 3035. The sum of the electricity storage capacity of pumped storage hydropower plants within the NUTS 2 areas is the sum of the storage capacity of the HPHS labelled hydropower plants located within the NUTS 2 areas. For HPHS labelled hydropower plants whose storage capacity is not given or given as zero, the storage capacity is assumed to be six times the installed power capacity of the plant. The last set of data attributed to NUTS 2 area dataset is the electricity generation potential from wind and solar technology available in the NUTS 2 areas. Two factors determine the electricity generation potential. The first factor is the installation capacity potential assigned to that region which is dependent on the eligible land or offshore area assigned to the NUTS 2 area.

The Geospatial Land Availability for Energy Systems framework (GLAES) [8] is the tool used to determine the eligible areas. The eligible land areas are calculated by applying the med scenario from [8] on the NUTS 2 area geometry. The eligible offshore areas within the exclusive economic zones are determined by eliminating non-eligible areas. These non-eligible areas are areas listed in the World Database on Protected Areas [7], areas with more than 60 m water depths, areas wherein 2017 ships were recorded to have spent in average more than one hour in a square km per month, areas within 12 nautical miles of the coast, areas within one nautical mile of gas and oil pipelines [6]. 30% of the remaining eligible area is proportionally distributed to NUTS 2 areas according to the size of their coastal borders to their exclusive economic zone. The installation capacity potential of offshore wind, onshore wind and solar are calculated using the capacity density values 5.36 MW/km^2^, 4 MW/km^2^ and 12 MW/km^2^ respectively. The second factor used to determine the electricity generation potential, of wind and solar, is the average full load hours. The full load hours of technology is the annual sum of the normalised maximum power output profile assigned to the NUTS 2 area taken from the renewables.ninja platform [13]. The full load hours of the onshore wind and solar are multiplied with their respective potential installed capacities to calculate their electricity generation potential within a NUTS 2 area. The offshore wind electricity generation potential is calculated by multiplying the full load hours with the potential offshore capacity assigned to a NUTS 2 areas. The technology with the highest electricity generation potential is assigned to the NUTS 2 area dataset.

## Grouping island NUTS 2 areas

Certain countries have NUTS 2 areas that are islands and thus do not share any vertexes with other NUTS 2 areas. The max-p-regions method has a contiguity constraint that ensures all NUTS 2 areas within a region shares at least one vertex with another NUTS 2 area in the same region. To enable that island NUTS 2 areas are capable of joining with other NUTS 2 areas to build regions they are grouped with the nearest continental NUTS 2 area. The geometries of these grouped NUTS 2 areas are joined to construct a single geometry. Table 1 has details of the islands and the continental NUTS 2 areas.

**Table 1:** The island NUTS 2 areas and their respective continental NUTS 2 area to which they are attached.

Groups	Island NUTS 2 areas	Continental NUTS 2 area
1	UKN0	UKM3
2	ITG2, ITG1	ITG3
3	FI20	FI1B
4	DK01, DK02	DK03

## Defining regions using the max-p-regions method

The Python Spatial Analysis Library (PySAL) contains a spatial optimisation library called spopt [14]. As described in the pseudocode 1 below, the *MaxPHeuristic* function of the spopt library, in conjunction with the libpysal python library, is used to implement the max-p-regions method.

**Fig. 1:**
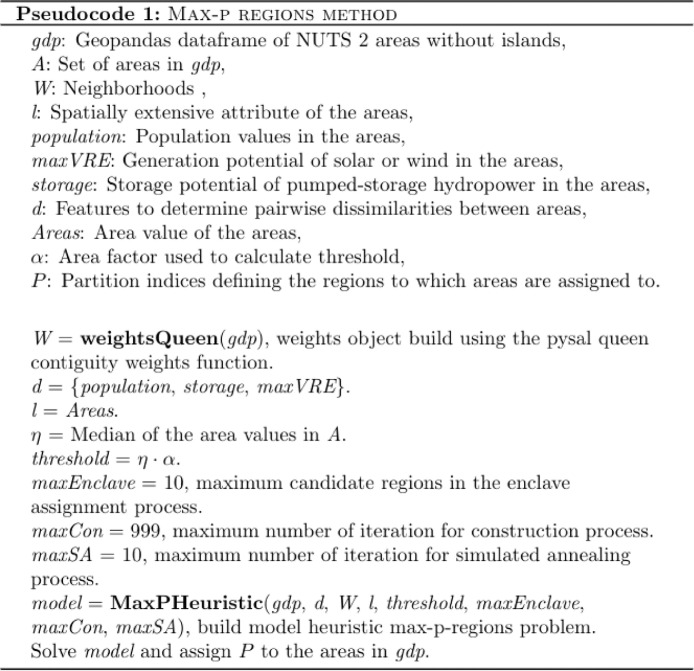
Pseudocode detailing the parameters and functions used to conduct the max-p regions method.

The *MaxPHeuristic* function uses a heuristic approach of solving the max-p-regions problem which defines an objective function and constraints used to maximise the heterogeneity between the regions and maximise the number of regions created. As presented in [15], there are three options to conduct a local search when finding the best feasible solution to define regions. The *MaxPHeuristic* function uses the simulate annealing approach. A contiguity constraint is defined in the *MaxPHeuristic* function by assigning weight values to the NUTS 2 areas using the queen contiguity weights function provided by libpysal [16]. The max-p regions method is detailed in Fig. 1.

The result of the *MaxPHeuristic* function returns an assigned partition value for each NUTS 2 area. The NUTS 2 areas that were assigned the same partition value are grouped into the same region. The size and number of regions created depend on the threshold value. By changing the area factor used to determine the threshold value, it is possible to vary the size and number of regions allows for the variation of the spatial scale of the energy system model. The area factor must be less than the fraction of the sum of the area values to the median of the area values of the NUTS 2 areas.

The energy-related max-p-regions method defines regions of NUTS 2 areas based on their energy-related spatial attributes. The aim of the method is to differentiate regions according to how they consume, produce and store electricity in a power system with high wind and solar energy penetration. Therefore three spatial attributes used to define heterogeneity of the NUTS 2 areas are population; wind and solar resource potential; and pumped-hydro storage capacity. These three spatial attributes are assigned as the features attribute in the *MaxPHeuristic* function to create energy-related max-p regions.

**Fig. 2:**
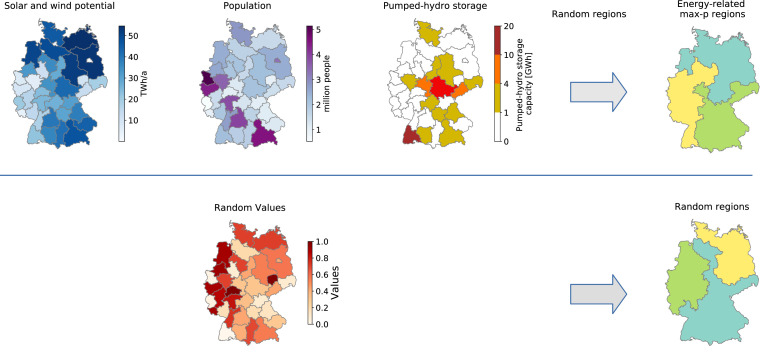
Example of creating three energy-related max-p regions and three random regions from the NUTS 2 areas of Germany. The energy-related attributes solar and wind potential, population and pumped-hydro storage are used to determine the heterogeneity between regions and create energy-related max-p regions. Random values from 0 to 1 and are used to determine the heterogeneity between regions to create random regions.

An alternative method to define regions is to associate NUTS 2 areas at random to regions while maintaining the contiguity of the regions, hereafter referred to as the random-regions method. The random-regions method assigns a set of unique random values from 0 to 1 to the NUTS 2 areas. The random-regions method follows the pseudocode 1 in Fig. 1 but uses the random values instead of the energy-related spatial attributes to determine the heterogeneity between regions when creating the model with the *MaxPHeuristic* function. The random-regions method was used in [17] to investigate the effectiveness of the max-p regions method to minimise the effects of spatial resolution reduction on power system models. The NUTS 2 area spatial dataset can be filtered to focus on a particular set of NUTS 2 areas. The NUTS 2 areas of Germany were filtered to create energy-related regions and random regions depicted in Fig. 2.

**Fig. 3:**
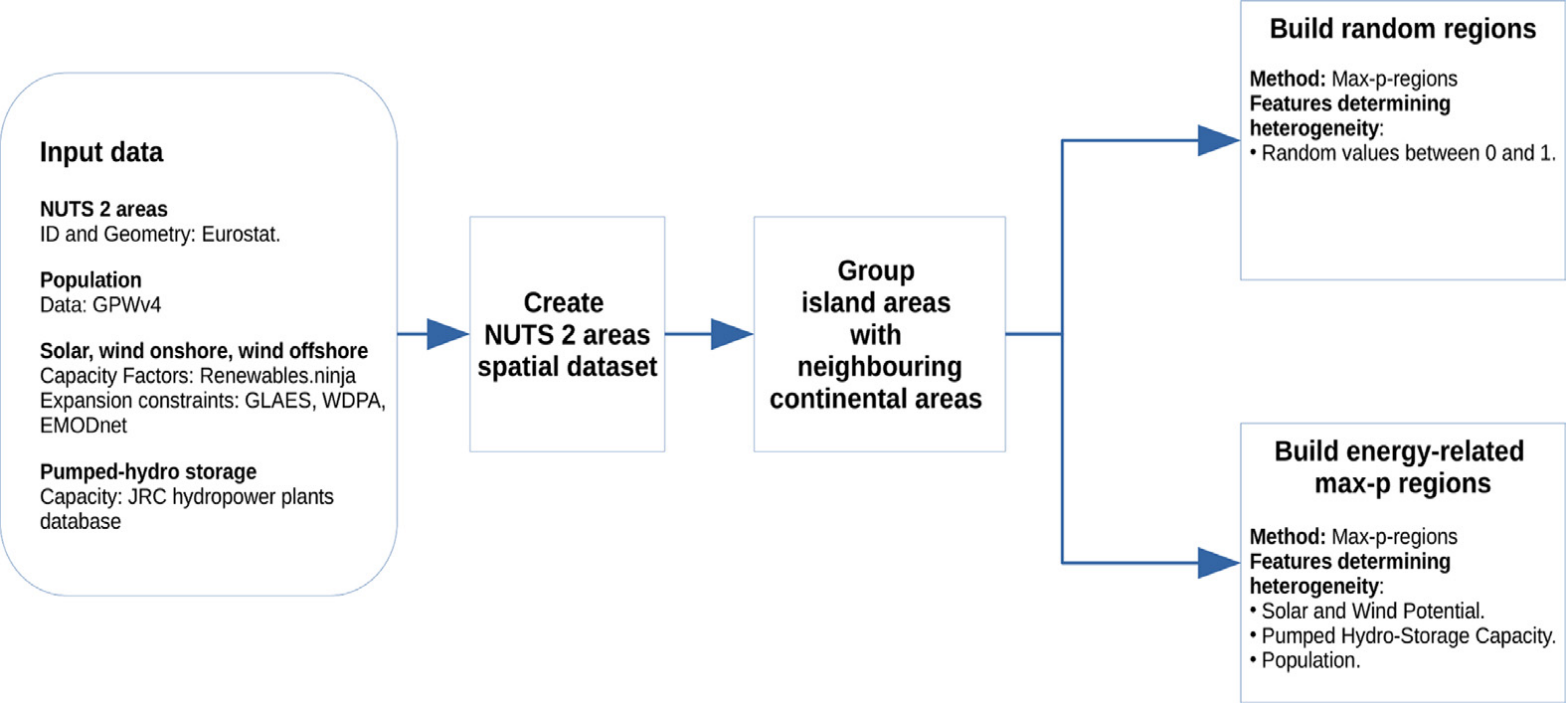
Illustration of the process to create energy-related max-p regions and random regions.

The complete process to create the energy-related max-p regions and the random regions is illustrated in Fig. 3.

## Conclusion

This article provides a detailed description of how to define regions using the max-p regions method. The method can help minimise the effects of spatial resolution reduction on an energy system model. The max-p regions method can be improved with more spatial data availability on the onshore and offshore area to more accurately determine the potential of wind and solar within the areas.

## Declaration of Competing Interest

The author confirms that there are no conflicts of interest.
